# Genetic structure of Mount Huang honey bee (*Apis cerana*) populations: evidence from microsatellite polymorphism

**DOI:** 10.1186/s41065-016-0010-4

**Published:** 2016-07-06

**Authors:** Fang Liu, Tengfei Shi, Sisi Huang, Linsheng Yu, Shoudong Bi

**Affiliations:** 1 Honey bee Research Institute, College of Animal Science & Technology, Anhui Agricultural University, Hefei, 230036 China; 2 College of Forest and Garden, Anhui Agricultural University, Hefei, 230000, 230036 Anhui China; 3 Apiculture Research Institute, College of Animal Science & Technology, Anhui Agricultural University, Hefei, 230036 Anhui China

**Keywords:** *Apis cerana*, Honey bee, Microsatellite, Mount huang, SSRs

## Abstract

**Background:**

The Mount Huang eastern honey bees (*Apis cerana*) are an endemic population, which is well adapted to the local agricultural and ecological environment. In this study, the genetic structure of seven eastern honey bees (*A. cerana*) populations from Mount Huang in China were analyzed by SSR (simple sequence repeat) markers.

**Results:**

The results revealed that 16 pairs of primers used amplified a total of 143 alleles. The number of alleles per locus ranged from 6 to 13, with a mean value of 8.94 alleles per locus. Observed and expected heterozygosities showed mean values of 0.446 and 0.831 respectively. UPGMA cluster analysis grouped seven eastern honey bees in three groups.

**Conclusion:**

The results obtained show a high genetic diversity in the honey bee populations studied in Mount Huang, and high differentiation among all the populations, suggesting that scarce exchange of honey bee species happened in Mount Huang. Our study demonstrated that the Mount Huang honey bee populations still have a natural genome worth being protected for conservation.

**Electronic supplementary material:**

The online version of this article (doi:10.1186/s41065-016-0010-4) contains supplementary material, which is available to authorized users.

## Background

Mount Huang lies in the southern end of the Anhui province of China (Wannan), between 117°02′–118°55′ east longitude and 29°24′–30°24′ north latitude. It has a typical humid subtropical monsoon climate, luxuriantly diverse flora and fauna, including honey bees. The Mount Huang eastern honey bees (*A. cerana*) are an endemic population, which is well adapted to the local agricultural and ecological environment. They have a particularly strong foraging instinct and resistance to parasitic mites as well as American foulbrood. This endemic population was listed in the first directory of potential breeding stock for conservation in 2001. The *A. cerana* conservation document Wannan (Wan D/XM01-19-87) was published according to ecological standards in Anhui province. However, since then, a massive replacement of the endemic honey bees has occurred, especially after the importation of *Apis mellifera* [1]. In some regions, this has resulted in almost complete replacement of endemic *A. cerana* populations with imported stocks.

Microsatellites are suitable nuclear markers used in population genetic studies in various taxonomic groups [2, 3]. This is due to their mass polymorphic loci, codominance, abundance throughout the genome, and relative ease of scoring [4]. The genetic structure of eastern honey bee populations from many regions of China have been analyzed using microsatellite markers. These regions mostly include areas in the middle east (Fujian, Jiangsu and Zhejiang provinces) and the south west (Tibei, Yunnan province and Hainan province). Zhu et al. [5] used five microsatellite loci to analyze the genetic structure of six *A. cerana* populations from Fujian province, their results showed that the mean expected heterozygosities (*He*) of populations from most of regions in Fujian was 0.5171 . In another study, a significant difference in genetic structures was found among honey bees from six regions in Wuyi Mountain located in Fujian province, the mean *He* of the six different region population was range from 0.4525–0.5613 [6]. Using six microsatellite loci, Ji et al. [7] found higher population heterozygosity and abundant genetic diversity in *Apis cerana* as well as in *Apis mellifera* (western European honey bee) populations in Jiangsu province, they found the mean *He* of *A. cerana* population in this province was 0.4424. Cao et al. [8] analyzed the genetic structure of more than ten *A. cerana* populations from different regions in Zhejiang province, their results showed that the genetic diversity of *A. cerana* in Zhejiang was relatively low, with the mean *He* was 0.3179. Guo et al. [9] used 8 microsatellite markers found the *A. cerana* populations in Qinling-Data mountain areas has rich genetic polymorphism, with the mean *He*, number of alleles, polymorphism information content (*PIC*) were 0.6877, 7.71, 3.7488 respectively. Ji et al. [10] found significant differentiation among 20 *A. cerana* populations from geographically different regions in China using 21 microsatellite loci. 502 alleles were found, and the average value of *He* of 21 microsatellites was 0.8689. Additionally, the mean *He* of the populations from Wuding (Yunnan province), Hainan, Bomi Tibet was 0.695, 0.6641 and 0.562 respectively, revealing their relatively high genetic diversity. Genetic diversity of *A. cerana* populations from Lushui in Yunnan province was evaluated with 21 microsatellite markers. 121 alleles were found, and the mean number of effective alleles was 5.76, the average *He* and *PIC* of all loci were 0.6752 and 0.6192, respectively, these results indicated the high level of genetic diversity in Lushui [11]. Six *A. cerana* populations distributed in different geographic areas in the south west Yunnan province were also surveyed using 21 microsatellite loci. All populations showed high levels of heterozygosity except the population from Diqing, the average *He* of the total populations was 0.739, and a significant genetic differentiation was found among all six populations [12]. Taken together, the genetic variation of *A. cerana* populations is overall higher in the southwest of China than in the middle east of China.

However, information about genetic variation of *A. cerana* in Mount Huang of Anhui province, in south east China, is presently not available. Therefore, the objectives of this study were to examine the genetic structure of Mount Huang honey bee populations using microsatellite analyses. Our findings provide important information that can be used for effective management and conservation of *A. cerana* in Wannan.

## Methods

### Samples

Adult workers from 190 colonies were collected from seven locations covering the main bee keeping areas in Huangshan [13], south of Anhui province in China (Wannan). Over 25 colonies were obtained from Jing County (JC), Huangshan District (HSD), Qimen County (QMC), and Yi County (YC), whereas 30 colonies were collected from Jixi County (JXC), She County (SC) and Huizhou District (HZD). The distances between the locations are more than 10 km. Ten bees from each colony were subjected to genetic analysis. Honey bee samples were preserved in 95 % ethanol and kept at −20 °C until needed.

### DNA extraction and microsatellites amplification

Total DNA was extracted from the thorax of each bee, following the method of Smith and Hagen [14], and the concentration was then estimated using ultraviolet spectrophotometer MV-2201. DNA was stored at −20 °C until needed. The primer sequences of these loci were as previously described by Solignac et al. [15]. The annealing temperature and the concentration of MgCl_2_ are listed in Table 1. The PCR conditions were as follow: one cycle at 95 °C for 5 min; 35 cycles of 95 °C for 45 s, 50–60 °C for 45 s, 72 °C for 60 s; and one cycle at 72 °C for 8 min. The PCR products were electrophoretically fractionated in 6% denaturating polyacrylamide gels and visualized by silver staining. The size of amplified microsatellites was measured using EDA20 Kodak imaging system (Eastman Kodak, USA).

**Table 1:** Location of 16 microsatellite loci in chromosome or linkage group and PCR conditions

Locus	Chromosome or linkage group	GenBank Accession number	M_g_^2+^ (mmol/L)	Annealing temperature (°C)
AP243	ChrLG1	AJ509466	2.2	57.5
A043	ChrLG1	AJ509256	1.5	55
AP274	ChrLG3	AJ509486	2.0	55.0
AT105	ChrLG5	AJ509553	2.2	56.5
A113	ChrLG6	AJ509290	2.0	58.2
A107	ChrLG7	AJ509287	2.0	57.0
A024d	ChrLG7	AJ509241	2.0	57.0
A014	ChrLG8	AJ509239	2.0	55.6
A088	ChrLG8	AJ509283	1.6	58.0
A007	ChrLG8	AJ509236	2.0	55
AP033	ChrLG10	AJ509319	2.2	56.5
AP297	ChrLG12	AJ509499	1.0	54
A029	ChrLG12	AJ509245	1.2	58
A035	ChrLG14	AJ509251	2.0	53.4
A028	ChrLG14	AJ509244	2.0	55.0
AG005C	ChrLG16	AJ509723	2.0	55.0

### Statistical analyses

The number of alleles, allele frequencies, observed and expected heterozygosity (*Ho* and *He*) of each microsatellite locus were computed with Microsatellite-Toolkit software [16]. Genetic and genotypic differentiation tests were conducted among the 7 populations using GENEPOP 4.1. The exact test for Hardy-Weinberg equilibrium (HWE), genetic structure and gene flow (Nm) were calculated with GENEPOP 4.1 [17]. F-statistics between populations were calculated using the software FSTAT (v2.9.3) [18]. The significance level of multiple comparisons was calculated using the sequential Bonferroni procedure [19].

The number and frequency of alleles were used to obtain the Da genetic distance between each population. Population tree based on Da values was constructed using the neighbor-joining (NJ) algorithms and unweighted pair group method with arithmetic mean (UPGMA) using PHYLIP software package (version 3.69) [20].

## Results

The locations where honey bee samples were collected were mentioned in Yu et al. [13]. The number and size of alleles, the value of *Ho* and *He* are listed in Additional file 1: Table S1. A total of 143 alleles were detected at 16 microsatellite loci. The overall parameters per population are shown in Additional file 1: Table S1. The gene diversity (the mean *He*) varied between 0.597 ± 0.109 in the HSD population and 0.71 ± 0.056 in the SC population, and mean observed heterozygosities between 0.398 ± 0.186 in HSD population and 0.495 ± 0.235 in JC population. The observed heterozygosity (*Ho*) was relatively high in all populations except that of JC at loci A035, and HSD population at loci A107. The mean number of alleles for each population ranged from 2.936 ± 0.853 in HSD to 6.438 ± 1.632 in SC population.

We detected significant deviations (*P* < 0.05) from Hardy-Weinberg equilibrium in 86 out of 112 population-locus combination and all deviations were in favor of homozygotes (Table 2). Both genic and genotypic differentiation tests showed highly significant (*P* < 0.0001) differentiation among all populations. Multilocus F_IS_ values varied between 0.181 (A029) and 0.618 (A088), while the F_IT_ values varied from 0.314 (A028) to 0.701 (AP297). A high heterozygote deficiency within populations and the total population were revealed by positive F_IS_ and F_IT_ (Table 3). The value of F_ST_ for the total populations was 0.171, all loci contributed significantly to the differentiation (*P <* 0.001).

**Table 2:** The *P* -value of every population and every locus in Hardy-Weinberg test

locus	*P* value						
	JC	HSD	JXC	YC	QMC	SC	HZD
AP243	0.4447	0.2713	0	0.0087	0.2283	0.0084	0.0034
A043	0.0001	0.0046	0	0	0.3076	0	0.0008
AP274	0.0003	0.0995	0.001	0.0058	0.2845	0	0.0017
AT105	0.003	0.3695	0.0002	0.071	0.0716	0	0.0813
A113	1	0	0	0.0023	0.0725	0	0.0025
A107	0.1234	0	0.1747	0.322	0.0402	0	0.7541
A024d	0.0001	0	0.0019	0.0027	0.0106	0	0.0009
A014	0.0001	0.0092	0	0.0117	0	0.0004	0
A088	0.0115	0.0001	0	0	0.0001	0	0
AP033	1	0.0025	0.0029	0.0034	0.0057	0.007	0.2327
AP297	0.0052	0.7253	0	0	0.0018	0.0001	0
A029	0.0243	0.9913	0.0195	0.8768	0.9816	0.1932	0
A007	0.0148	0.0001	0.0033	0.001	0.0027	0	0
A035	0	0.9728	0	0.101	0.0111	0	0.0026
A028	0.3279	0.0358	0.309	0.0004	0	0	0.0008
AG005C	0.0009	0	0.0014	0.0023	0.2447	0	0.0012

**Table 3:** F. coefficients of total of populations including 7 *A. cerana* in Mount Huangshan of Wannan

Locus	*F*_*IT*_	*F*_*ST*_	*F*_*IS*_
AP243	0.361***	0.108***	0.283***
A043	0.631***	0.203***	0.541***
AP274	0.453***	0.131***	0.372***
AT105	0.390***	0.134***	0.295***
A113	0.361***	0.126***	0.268***
A107	0.360***	0.198***	0.200***
A024d	0.527***	0.110***	0.470***
A014	0.568***	0.170***	0.479***
A088	0.681***	0.166***	0.618***
AP033	0.349***	0.181***	0.208***
AP297	0.701***	0.268***	0.589***
A029	0.404***	0.272***	0.181***
A007	0.444***	0.077***	0.398***
A035	0.604***	0.277***	0.458***
A028	0.314***	0.116***	0.225***
AG005C	0.481***	0.150***	0.390***
Mean ± SD	0.47 ± 0.032***	0.171 ± 0.016***	0.369 ± 0.035***

Note: Mean estimates from jack-knife over loci, standard deviations (SD) are given in parentheses; ***p < 0.001

**Fig. 1:**
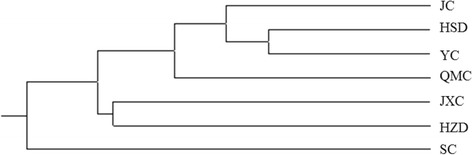
Dendogram of phylogenetic relationships among 7 *Apis cerana* populations based on Nei’s genetic distance using neighbor-joining method

Genetic distances between pairs of Mount Huang *A. cerana,* in the present study, were 0.2104-0.5027 (Table 4, lower triangle). The pairwise Nm value (Table 4, upper triangle) between populations varied between 0.6049 (SC and JXC) and 0.8345 (YC and HSD). The neighbor-joining tree (Fig. 1) was constructed according to the Da genetic distance between each pair of populations. The SC population formed a group separating from other populations. JXC and HZD formed a cluster, JC, HSD, YC and QMC grouped together.

**Table 4:** Nei’s genetic distance, Da (lower triangle) and the gene flow, Nm (up triangle) between populations

Population	JX	HSQ	JXX	YX	QMX	SX	HZQ
JX	–––––	0.7812	0.7326	0.8103	0.7714	0.6623	0.7503
HSQ	0.2469	–––––	0.6594	0.8345	0.6827	0.6053	0.6148
JXX	0.3112	0.4164	–––––	0.7201	0.6678	0.6049	0.7016
YX	0.2104	0.1809	0.3284	–––––	0.8050	0.6749	0.7239
QMX	0.2595	0.3817	0.4038	0.2169	–––––	0.6335	0.6568
SX	0.4120	0.5020	0.5027	0.3932	0.4565	–––––	0.6456
HZQ	0.2872	0.4864	0.3544	0.3231	0.4204	0.4375	–––––

The line means blank. There is no genetic distance between the same populations

## Discussion

Population differentiation was detected in Mount Huangshan *A. cerana* through analysis of microsatellite polymorphism. There are limited population genetic studies of Mount Huangshan *A. cerana*, using microsatellite analysis. A previous study by Yu et al. [13], using morphometric analysis reported similar finding. Therefore, our study adds to the knowledge of the genetic changes that may be occurring in this important endemic honey bee population. This knowledge can be used for genetic management and conservation efforts, for the preservation of this population, which was first listed in the directory of potential breeding stock for conservation in 2001. The pure *A. cerana* population has several desirable advantages biological features, including disease resistance and the ability to get rid of bee mites from colonies. To date, other studies of *A. cerana* by microsatellite loci have focused on the eastern and southern populations of China. According to those reports, the expected heterozygosity levels were highest among honey bee populations in the south west region, which ranged between 0.5625 and 0.738 [10–12], and were lowest among honey bees in the middle east reported as 0.3179-0.5613 [5–8]. In the present study, the microsatellite mean expected heterozygosity values for ranged between 0.597 and 0.781, which is similar to those of honey bees in Yunnan. This similarity may be attributable to similar geographic environments in Yunnan and Mount Huang, which are both mountainous with high altitude.

The microsatellite data also showed that, the Mount Huang populations seem to be more variable compared to those in the eastern Chinese region. Average allele numbers and heterozygosities were higher in Mount Huang populations than in Fujian [5, 6], Jiangsu [7], Zhejiang [8]. This difference may, in part, be attributable to differences in the evaluated population sizes.

Significant deviations from Hardy-Weinberg equilibrium were observed at loci A024d, A014, A088, and A007 in all investigated populations even with correction by sequential Bonferroni method. Such deviation may be due to miscoring of alleles resulting from the existence of shatter bands [21]. However, in this case it is unlikely this is the cause, because the band intensity among a group of stutter bands was very carefully examined in order to obtain consistent allele designation. Due to the use of heterospecific primers in this species, the other factor may be a result of the presence of null alleles at these microsatellite loci [22]. It will be premature to conclude which of these factors contribute heterozygote deficiencies of *A. cerana* in Mount Huang until these bees are examined using microsatellite primers developed from *A. cerana* rather than those from *A. mellifera* [23].

Considering the F_ST_ estimates, all 16 microsatellite loci contributed mostly to differentiate the populations (Table 3), corresponding to the low Nm value (Nm < 1) between populations, which indicated. The F_ST_ value of AP033 loci here is similar to Ji et al. [7]. The bee samples we collected in present study included wild honey bees and domestic honey bees, which also may contribute to the large differentiation among honey bees from different locations. A total of seven *Apis cerana* populations in Huangshan were isolated by high mountains [24], it is unlikely gene flow for these honey bees.

Large genetic distances were observed among all populations, especially those between *A. cerana* from SC population and other populations (*d* = 0.3932-0.5027). The seven *A. cerana* populations in this study could then be genetically divided into three different groups, which included one group with JC, HSD, YC, and QMC populations, another with JXC and HZD populations, while the SC population was a separate group (Fig. 1). YC was firstly linked to HSD, which was also indicated by the relative high Nm values (0.7812) between them. While SC formed one group alone, it is located between Mount Huang and Mount Tianmu, this may have been an important barrier to gene flow for SC *A. cerana* and other populations.

## Conclusion

Microsatellite data obtained in this study demonstrated that the Mount Huanghoney bee populations still have a natural genome worth being protected for conservation. However, further screening of *A. cerana* on a larger geographic scale should be carried out to fully elucidate the patterns of conspecific and intraspecific population structure in Wannan *A. cerana*.

## Additional file

Additional file 1: Table S1. Allele frequencies, proportion of observed (*Ho*) and expected (*He*) heterozygosity of 16 microsatellites loci across seven populations of Mount Huangshan *A. cerana*, n is the number of individual bees examined. (DOC 218 kb)
