# Supplementary material for: Genetic structure of Mount Huang honey bee (Apis cerana) populations: evidence from microsatellite polymorphism
Source: Hereditas. 2016 Jul 6;153:8. doi: 10.1186/s41065-016-0010-4 (PMC5226090; doi:10.1186/s41065-016-0010-4)
Supplement: Additional file 1: Table S1. — Allele frequencies, proportion of observed (Ho) and expected (He) heterozygosity of 16 microsatellites loci across seven populations of Mount Huangshan A. cerana, n is the number of individual bees examined. (DOC 218 kb) [file 41065_2016_10_MOESM1_ESM.doc]

**Supplementary table 1.** Allele frequencies, proportion of observed (*Ho*) and expected(*He*) heterozygosity of 16 microsatellites loci across seven populations of Mount Huangshan *A. cerana*, n is the number of individual bees examined.

|  | Allele  (bp) | JC  (n=25) | HSD  (n=25) | JXC  (n=30) | YC  (n=25) | QMC  (n=25) | SC  (n=30) | HZD  (n=30) |
| --- | --- | --- | --- | --- | --- | --- | --- | --- |
| AP243 | 116 | 0.12 | 0 | 0 | 0 | 0 | 0.05 | 0 |
|  | 160 | 0 | 0 | 0.217 | 0 | 0 | 0 | 0 |
|  | 169 | 0.12 | 0.2 | 0.25 | 0 | 0.2 | 0.033 | 0.283 |
|  | 185 | 0.26 | 0.16 | 0 | 0 | 0.08 | 0 | 0 |
|  | 190 | 0 | 0 | 0 | 0.1 | 0 | 0.067 | 0 |
|  | 255 | 0 | 0 | 0.067 | 0 | 0 | 0.117 | 0.067 |
|  | 267 | 0.24 | 0.48 | 0.117 | 0.36 | 0.62 | 0.467 | 0.35 |
|  | 267 | 0 | 0 | 0 | 0 | 0.1 | 0.217 | 0.133 |
|  | 272 | 0.26 | 0 | 0.35 | 0.3 | 0 | 0 | 0.167 |
|  | 300 | 0 | 0.16 | 0 | 0.24 | 0 | 0.05 | 0 |
| Number of alleles | | 5 | 4 | 5 | 4 | 4 | 7 | 5 |
| Ho | | 0.76 | 0.56 | 0.4 | 0.44 | 0.52 | 0.533 | 0.467 |
| He | | 0.778 | 0.678 | 0.750 | 0.713 | 0.560 | 0.711 | 0.747 |
| A043 | 118 | 0.4 | 0 | 0 | 0 | 0 | 0.267 | 0 |
|  | 130 | 0.32 | 0 | 0 | 0 | 0 | 0 | 0.317 |
|  | 140 | 0 | 0.16 | 0.233 | 0.46 | 0.78 | 0.25 | 0.267 |
|  | 144 | 0 | 0.2 | 0.317 | 0.16 | 0 | 0.15 | 0.25 |
|  | 155 | 0.28 | 0.64 | 0.283 | 0.38 | 0 | 0.333 | 0 |
|  | 252 | 0 | 0 | 0 | 0 | 0.22 | 0 | 0 |
|  | 350 | 0 | 0 | 0.167 | 0 | 0 | 0 | 0.167 |
| Number of alleles | | 3 | 3 | 4 | 3 | 2 | 4 | 4 |
| Ho | | 0.32 | 0.32 | 0.367 | 0.2 | 0.28 | 0.167 | 0.433 |
| He | | 0.659 | 0.525 | 0.737 | 0.618 | 0.343 | 0.733 | 0.738 |
| AP274 | 90 | 0 | 0 | 0.433 | 0 | 0 | 0.167 | 0 |
|  | 104 | 0.28 | 0.46 | 0 | 0.36 | 0.18 | 0.233 | 0.117 |
|  | 110 | 0.4 | 0.32 | 0 | 0.3 | 0 | 0.117 | 0.133 |
|  | 124 | 0.32 | 0.22 | 0.467 | 0.34 | 0.6 | 0.483 | 0.4 |
|  | 146 | 0 | 0 | 0.1 | 0 | 0 | 0 | 0.05 |
|  | 267 | 0 | 0 | 0 | 0 | 0 | 0 | 0.3 |
|  | 297 | 0 | 0 | 0 | 0 | 0.22 | 0 | 0 |
| Number of alleles | | 3 | 3 | 3 | 3 | 3 | 4 | 5 |
| Ho | | 0.32 | 0.52 | 0.367 | 0.44 | 0.56 | 0.267 | 0.467 |
| He | | 0.659 | 0.638 | 0.585 | 0.665 | 0.559 | 0.671 | 0.716 |
| AT105 | 130 | 0.34 | 0 | 0 | 0 | 0 | 0.1 | 0.183 |
|  | 162 | 0.22 | 0.22 | 0.383 | 0 | 0.5 | 0.367 | 0.333 |
|  | 174 | 0.06 | 0 | 0.15 | 0.44 | 0.42 | 0.067 | 0.283 |
|  | 180 | 0.12 | 0 | 0 | 0 | 0 | 0 | 0 |
|  | 188 | 0.12 | 0.4 | 0.25 | 0.56 | 0.08 | 0.25 | 0.2 |
|  | 258 | 0 | 0 | 0.217 | 0 | 0 | 0.15 | 0 |
|  | 290 | 0.14 | 0.38 | 0 | 0 | 0 | 0.067 | 0 |
| Number of alleles | | 6 | 3 | 4 | 2 | 3 | 6 | 4 |
| Ho | | 0.56 | 0.6 | 0.467 | 0.32 | 0.36 | 0.467 | 0.633 |
| He | | 0.784 | 0.647 | 0.721 | 0.493 | 0.567 | 0.761 | 0.736 |
| A113 | 110 | 0.32 | 0 | 0.15 | 0.32 | 0.08 | 0.15 | 0 |
|  | 125 | 0 | 0 | 0 | 0 | 0 | 0.217 | 0 |
|  | 147 | 0 | 0.26 | 0.1 | 0 | 0 | 0.033 | 0 |
|  | 174 | 0 | 0 | 0.033 | 0 | 0 | 0.033 | 0 |
|  | 192 | 0.14 | 0.34 | 0.55 | 0.24 | 0.52 | 0.083 | 0.617 |
|  | 213 | 0.06 | 0 | 0 | 0.2 | 0.22 | 0.083 | 0.05 |
|  | 217 | 0.22 | 0 | 0.167 | 0.24 | 0.18 | 0.133 | 0.117 |
|  | 223 | 0.26 | 0.4 | 0 | 0 | 0 | 0.183 | 0.1 |
|  | 238 | 0 | 0 | 0 | 0 | 0 | 0.083 | 0.117 |
| Number of alleles | | 5 | 3 | 5 | 4 | 4 | 9 | 5 |
| Ho | | 1 | 0.24 | 0.367 | 0.52 | 0.56 | 0.6 | 0.4 |
| He | | 0.758 | 0.657 | 0.636 | 0.742 | 0.642 | 0.856 | 0.579 |
| A107 | 90 | 0 | 0 | 0 | 0 | 0.38 | 0 | 0.15 |
|  | 160 | 0.24 | 0 | 0 | 0 | 0 | 0.25 | 0 |
|  | 170 | 0.12 | 0.32 | 0 | 0.42 | 0.14 | 0.167 | 0.067 |
|  | 180 | 0 | 0 | 0.25 | 0 | 0.48 | 0.1 | 0.25 |
|  | 184 | 0.2 | 0 | 0.2 | 0 | 0 | 0.083 | 0.35 |
|  | 190 | 0 | 0.68 | 0.25 | 0.32 | 0 | 0.083 | 0 |
|  | 217 | 0.14 | 0 | 0 | 0 | 0 | 0.067 | 0 |
|  | 238 | 0.16 | 0 | 0 | 0 | 0 | 0.083 | 0 |
|  | 252 | 0.14 | 0 | 0 | 0.26 | 0 | 0.083 | 0 |
|  | 305 | 0 | 0 | 0.3 | 0 | 0 | 0.083 | 0.183 |
| Number of alleles | | 6 | 2 | 4 | 3 | 2 | 9 | 5 |
| Ho | | 0.76 | 0 | 0.7 | 0.64 | 0.52 | 0.533 | 0.8 |
| He | | 0.823 | 0.435 | 0.745 | 0.654 | 0.606 | 0.861 | 0.755 |
| A024d | 85 | 0 | 0 | 0 | 0 | 0.32 | 0.15 | 0 |
|  | 96 | 0.4 | 0 | 0.467 | 0.3 | 0 | 0.25 | 0.2 |
|  | 100 | 0 | 0 | 0.05 | 0 | 0 | 0 | 0 |
|  | 104 | 0.3 | 0.3 | 0.483 | 0.36 | 0.3 | 0.333 | 0.167 |
|  | 111 | 0.3 | 0.34 | 0 | 0.34 | 0.38 | 0.267 | 0.183 |
|  | 123 | 0 | 0.36 | 0 | 0 | 0 | 0 | 0.217 |
|  | 192 | 0 | 0 | 0 | 0 | 0 | 0 | 0.233 |
| Number of alleles | | 3 | 2 | 3 | 3 | 3 | 4 | 5 |
| Ho | | 0.28 | 0.24 | 0.333 | 0.4 | 0.44 | 0.3 | 0.567 |
| He | | 0.66 | 0.665 | 0.546 | 0.665 | 0.663 | 0.733 | 0.797 |
| A014 | 160 | 0 | 0 | 0.15 | 0 | 0 | 0 | 0 |
|  | 170 | 0 | 0 | 0 | 0.5 | 0.24 | 0 | 0.233 |
|  | 180 | 0.22 | 0.52 | 0.233 | 0 | 0 | 0.117 | 0.217 |
|  | 184 | 0 | 0.48 | 0 | 0.32 | 0.24 | 0.283 | 0.167 |
|  | 188 | 0.22 | 0 | 0 | 0 | 0.3 | 0.183 | 0.067 |
|  | 200 | 0 | 0 | 0.183 | 0.18 | 0.22 | 0.067 | 0 |
|  | 228 | 0.26 | 0 | 0 | 0 | 0 | 0.083 | 0 |
|  | 242 | 0 | 0 | 0.267 | 0 | 0 | 0.167 | 0 |
|  | 250 | 0.3 | 0 | 0 | 0 | 0 | 0.1 | 0 |
|  | 267 | 0 | 0 | 0.167 | 0 | 0 | 0 | 0.317 |
| Number of alleles | | 4 | 2 | 5 | 3 | 4 | 7 | 5 |
| Ho | | 0.4 | 0.24 | 0.367 | 0.36 | 0.28 | 0.567 | 0.433 |
| He | | 0.746 | 0.499 | 0.588 | 0.615 | 0.747 | 0.823 | 0.766 |
| A088 | 120 | 0.4 | 0.3 | 0 | 0 | 0 | 0.267 | 0 |
|  | 125 | 0 | 0.44 | 0 | 0 | 0.4 | 0.167 | 0 |
|  | 140 | 0 | 0 | 0 | 0.24 | 0.12 | 0.133 | 0 |
|  | 144 | 0 | 0 | 0 | 0.2 | 0.24 | 0.133 | 0.117 |
|  | 147 | 0.34 | 0 | 0 | 0.16 | 0.24 | 0.067 | 0.05 |
|  | 150 | 0 | 0.26 | 0.25 | 0.16 | 0 | 0.05 | 0.367 |
|  | 155 | 0 | 0 | 0.35 | 0 | 0 | 0 | 0.3 |
|  | 158 | 0.26 | 0 | 0.233 | 0 | 0 | 0.183 | 0.167 |
|  | 170 | 0 | 0 | 0.167 | 0.24 | 0 | 0 | 0 |
| Number of alleles | | 3 | 3 | 4 | 5 | 4 | 7 | 5 |
| Ho | | 0.44 | 0.28 | 0.167 | 0.16 | 0.4 | 0.267 | 0.333 |
| He | | 0.657 | 0.649 | 0.733 | 0.794 | 0.710 | 0.825 | 0.731 |
| AP033 | 90 | 0 | 0 | 0 | 0.22 | 0 | 0 | 0 |
|  | 115 | 0 | 0 | 0.15 | 0 | 0 | 0 | 0.183 |
|  | 190 | 0 | 0.4 | 0 | 0.26 | 0.22 | 0 | 0 |
|  | 192 | 0 | 0 | 0 | 0.08 | 0 | 0 | 0 |
|  | 214 | 0 | 0 | 0.117 | 0 | 0 | 0.117 | 0.167 |
|  | 220 | 0 | 0 | 0 | 0 | 0 | 0.317 | 0.1 |
|  | 224 | 0.7 | 0 | 0.117 | 0.24 | 0 | 0 | 0 |
|  | 234 | 0.3 | 0.28 | 0.083 | 0 | 0.2 | 0.033 | 0.1 |
|  | 240 | 0 | 0.16 | 0 | 0.2 | 0.2 | 0 | 0.1 |
|  | 255 | 0 | 0 | 0.217 | 0 | 0 | 0.15 | 0.167 |
|  | 269 | 0 | 0.16 | 0.15 | 0 | 0.38 | 0.15 | 0 |
|  | 289 | 0 | 0 | 0.167 | 0 | 0 | 0.233 | 0.183 |
| Number of alleles | | 2 | 4 | 7 | 5 | 4 | 6 | 7 |
| Ho | | 0.6 | 0.48 | 0.633 | 0.52 | 0.52 | 0.533 | 0.867 |
| He | | 0.42 | 0.710 | 0.846 | 0.78 | 0.727 | 0.785 | 0.847 |
| AP297 | 116 | 0.38 | 0 | 0 | 0 | 0 | 0.15 | 0 |
|  | 124 | 0.28 | 0.7 | 0 | 0.6 | 0.28 | 0.033 | 0.467 |
|  | 126 | 0 | 0 | 0.6 | 0 | 0.28 | 0.067 | 0.1 |
|  | 130 | 0 | 0 | 0.4 | 0 | 0 | 0.283 | 0.433 |
|  | 142 | 0.34 | 0.3 | 0 | 0.16 | 0 | 0.217 | 0 |
|  | 154 | 0 | 0 | 0 | 0.24 | 0.44 | 0.25 | 0 |
| Number of alleles | | 3 | 2 | 2 | 3 | 3 | 6 | 3 |
| Ho | | 0.44 | 0.44 | 0 | 0.16 | 0.4 | 0.333 | 0.067 |
| He | | 0.6616 | 0.42 | 0.48 | 0.5568 | 0.6496 | 0.782 | 0.584 |
| A029 | 108 | 0.44 | 0 | 0 | 0 | 0 | 0.467 | 0 |
|  | 123 | 0.38 | 0.42 | 0.067 | 0.66 | 0.3 | 0.217 | 0 |
|  | 138 | 0.18 | 0 | 0 | 0 | 0 | 0.117 | 0.283 |
|  | 147 | 0 | 0 | 0.05 | 0 | 0.58 | 0.067 | 0.167 |
|  | 154 | 0 | 0.58 | 0.283 | 0.34 | 0.12 | 0.067 | 0 |
|  | 242 | 0 | 0 | 0.267 | 0 | 0 | 0.067 | 0.45 |
|  | 270 | 0 | 0 | 0.333 | 0 | 0 | 0 | 0.1 |
| Number of alleles | | 3 | 2 | 5 | 2 | 3 | 6 | 4 |
| Ho | | 0.52 | 0.68 | 0.467 | 0.52 | 0.72 | 0.7 | 0.1 |
| He | | 0.630 | 0.487 | 0.731 | 0.449 | 0.559 | 0.708 | 0.680 |
| A007 | 80 | 0.12 | 0.12 | 0.417 | 0.32 | 0.18 | 0.067 | 0 |
|  | 89 | 0.44 | 0.44 | 0.283 | 0.52 | 0.58 | 0.267 | 0.383 |
|  | 100 | 0.2 | 0.14 | 0.033 | 0.16 | 0.24 | 0.117 | 0.1 |
|  | 110 | 0.24 | 0.3 | 0 | 0 | 0 | 0.233 | 0 |
|  | 123 | 0 | 0 | 0.067 | 0 | 0 | 0.2 | 0.083 |
|  | 230 | 0 | 0 | 0.133 | 0 | 0 | 0.1 | 0.15 |
|  | 234 | 0 | 0 | 0 | 0 | 0 | 0.017 | 0.133 |
|  | 260 | 0 | 0 | 0.067 | 0 | 0 | 0 | 0.15 |
| Number of alleles | | 4 | 4 | 6 | 3 | 3 | 7 | 6 |
| Ho | | 0.48 | 0.36 | 0.433 | 0.36 | 0.36 | 0.467 | 0.537 |
| He | | 0.694 | 0.682 | 0.718 | 0.602 | 0.574 | 0.806 | 0.774 |
| A035 | 90 | 0.2 | 0 | 0 | 0 | 0 | 0.133 | 0 |
|  | 99 | 0 | 0 | 0.217 | 0 | 0.18 | 0.267 | 0.217 |
|  | 104 | 0 | 0 | 0.283 | 0 | 0.18 | 0.2 | 0.233 |
|  | 114 | 0 | 0 | 0.15 | 0 | 0.24 | 0.1 | 0.55 |
|  | 123 | 0 | 0 | 0.233 | 0 | 0 | 0.05 | 0 |
|  | 150 | 0 | 0 | 0 | 0 | 0.4 | 0 | 0 |
|  | 174 | 0.28 | 0 | 0.05 | 0 | 0 | 0 | 0 |
|  | 200 | 0.52 | 0 | 0.067 | 0.22 | 0 | 0 | 0 |
|  | 223 | 0 | 0.68 | 0 | 0 | 0 | 0 | 0 |
|  | 230 | 0 | 0 | 0 | 0.56 | 0 | 0.25 | 0 |
|  | 241 | 0 | 0.32 | 0 | 0.22 | 0 | 0 | 0 |
| Number of alleles | | 3 | 2 | 6 | 3 | 4 | 6 | 3 |
| Ho | | 0 | 0.56 | 0.5 | 0.44 | 0.56 | 0.167 | 0.367 |
| He | | 0.611 | 0.435 | 0.789 | 0.590 | 0.718 | 0.796 | 0.596 |
| A028 | 108 | 0.42 | 0.4 | 0.033 | 0 | 0 | 0.233 | 0 |
|  | 114 | 0 | 0 | 0.217 | 0.16 | 0 | 0.3 | 0 |
|  | 120 | 0.2 | 0.14 | 0.15 | 0.04 | 0 | 0.1 | 0.367 |
|  | 125 | 0 | 0 | 0 | 0.06 | 0 | 0.067 | 0 |
|  | 135 | 0.18 | 0.3 | 0.05 | 0.16 | 0.24 | 0.05 | 0.067 |
|  | 140 | 0 | 0 | 0.15 | 0.12 | 0.12 | 0 | 0 |
|  | 145 | 0 | 0 | 0 | 0.08 | 0.28 | 0.067 | 0.1 |
|  | 150 | 0 | 0 | 0.133 | 0.16 | 0.16 | 0.067 | 0.117 |
|  | 154 | 0 | 0 | 0 | 0 | 0 | 0 | 0.083 |
|  | 162 | 0.2 | 0.16 | 0 | 0 | 0 | 0 | 0.067 |
|  | 174 | 0 | 0 | 0.267 | 0.12 | 0 | 0 | 0.067 |
|  | 220 | 0 | 0 | 0 | 0 | 0 | 0.067 | 0.133 |
|  | 267 | 0 | 0 | 0 | 0.1 | 0.2 | 0.05 | 0 |
| Number of alleles | | 4 | 4 | 7 | 9 | 5 | 9 | 8 |
| Ho | | 0.68 | 0.6 | 0.867 | 0.6 | 0.4 | 0.533 | 0.667 |
| He | | 0.711 | 0.705 | 0.815 | 0.873 | 0.784 | 0.823 | 0.804 |
| AG005C | 90 | 0 | 0 | 0.25 | 0.36 | 0 | 0 | 0.183 |
|  | 104 | 0 | 0 | 0.167 | 0.14 | 0 | 0.117 | 0.217 |
|  | 106 | 0 | 0.38 | 0 | 0 | 0 | 0.117 | 0 |
|  | 108 | 0.46 | 0 | 0 | 0 | 0 | 0 | 0 |
|  | 110 | 0 | 0 | 0 | 0.12 | 0.22 | 0.1 | 0.117 |
|  | 115 | 0.32 | 0.26 | 0 | 0 | 0.12 | 0.2 | 0 |
|  | 123 | 0.22 | 0.22 | 0 | 0.22 | 0 | 0.233 | 0.25 |
|  | 130 | 0 | 0.14 | 0.233 | 0.16 | 0.2 | 0.233 | 0.233 |
|  | 145 | 0 | 0 | 0.15 | 0 | 0.46 | 0 | 0 |
|  | 245 | 0 | 0 | 0.2 | 0 | 0 | 0 | 0 |
| Number of alleles | | 3 | 4 | 5 | 5 | 4 | 6 | 5 |
| Ho | | 0.36 | 0.24 | 0.567 | 0.52 | 0.68 | 0.333 | 0.567 |
| He | | 0.638 | 0.72 | 0.793 | 0.762 | 0.686 | 0.814 | 0.789 |
| Mean no. of alleles per locus | | 3.75±  1.146 | 2.936±  0.853 | 4.688±  1.401 | 3.75±  1.693 | 3.438±  0.814 | 6.438±  1.632 | 4.938±  1.289 |
| Average Ho  (SD) | | 0.50  (0.235) | 0.40  (0.186) | 0.44  (0.202) | 0.41  (0.147) | 0.47  (0.129) | 0.42  (0.162) | 0.48  (0.214) |
| Average He  (SD) | | 0.68  (0.094) | 0.60  (0.109) | 0.70  (0.104) | 0.66  (0.113) | 0.63  (0.107) | 0.78  (0.056) | 0.73  (0.08) |
